# A Comparative Study of Laparoscopic versus Open Pancreaticoduodenectomy for Ampulla of Vater Carcinoma

**DOI:** 10.3390/jcm9072214

**Published:** 2020-07-13

**Authors:** Daegwang Yoo, Ki Byung Song, Jong Woo Lee, Kyungyeon Hwang, Sarang Hong, Dakyum Shin, Dae Wook Hwang, Jae Hoon Lee, Woohyung Lee, Jaewoo Kwon, Yejong Park, Eunsung Jun, Song Cheol Kim

**Affiliations:** Division of Hepatobiliary and Pancreatic Surgery, Department of Surgery, Asan Medical Center, University of Ulsan College of Medicine, Seoul 05505, Korea; yoodaegwang@naver.com (D.Y.); hy_thename@hanmail.net (J.W.L.); hkyz84@gmail.com (K.H.); 8thofnovember@hanmail.net (S.H.); gracedkshin@gmail.com (D.S.); drdwhwang@gmail.com (D.W.H.); gooddr23@naver.com (J.H.L.); ywhnet@gmail.com (W.L.); skunlvup@naver.com (J.K.); blackpig856@gmail.com (Y.P.); eunsungjun@amc.seoul.kr (E.J.); drksc@amc.seoul.kr (S.C.K.)

**Keywords:** ampulla of Vater, cancer, laparoscopy, pancreaticoduodenectomy, propensity score

## Abstract

Several studies have compared laparoscopic pancreaticoduodenectomy (LPD) and open pancreaticoduodenectomy (OPD) in patients with periampullary carcinoma; however, only a few studies have made such a comparison on patients with ampulla of Vater cancer (AVC). We compared the perioperative and oncologic outcomes between LPD and OPD in patients with AVC using propensity-score-matched analysis. A total of 359 patients underwent PD due to AVC during the study period (76 LPD, 283 OPD). After propensity score matching, the LPD group showed significantly longer operation time than did the OPD group (400.2 vs. 344.6 min, *p* < 0.001). Nevertheless, the LPD group had fewer painkiller administrations (8.3 vs. 11.1, *p* < 0.049), fewer Grade II or more severe postoperative complications (15.9% vs. 34.8%, *p* = 0.012), and shorter postoperative hospital stays (13.7 vs. 17.3 days, *p* = 0.048), compared with the OPD group. There was no significant difference in recurrence-free outcomes and overall survival between the two groups (*p* = 0.754 and 0.768, respectively). Compared with OPD, LPD for AVC had comparative oncologic outcomes with less pain, less postoperative morbidity, and shorter hospital stays. LPD may serve as a promising alternative to OPD in patients with AVC.

## 1. Introduction

Ampulla of Vater cancer (AVC) is defined as a malignancy that arises within the ampullary complex, distal to the confluence of the distal common bile duct and the pancreatic duct. AVC is a rare malignancy and accounts for 0.2% of all digestive malignancies, with an annual incidence of 0.4–0.5 cases per 100,000 people per year [1,2].

Pancreaticoduodenectomy (PD) remains the mainstay curative treatment for patients with AVC, and owing to improvements in the technology and techniques of minimally invasive surgery, laparoscopic pancreaticoduodenectomy (LPD) is being performed more frequently in large volume hospitals. Following its first description by Gagner et al. in 1994 [3], LPD has been evaluated for its adequacy and feasibility in numerous studies [4,5,6,7,8,9,10,11,12,13,14]. Although LPD requires complex dissection and reconstruction, it offers acceptable perioperative and oncologic outcomes [4,5,6,7,9].

LPD is relatively easy to apply in AVC because AVC has little multivisceral invasion and a small tumor size. However, only a few reports have solely evaluated LPD for AVC [15,16], which included a small number of patients and lacked adequate matched groups that received open PD (OPD) for comparison [15]. In the present study, we retrospectively reviewed the oncologic and perioperative outcomes of AVC following LPD and OPD performed at a single large volume center and performed propensity score matching in order to compare the two surgical methods in terms of oncologic adequacy and perioperative outcomes.

## 2. Methods

### 2.1. Patient Database

Data from all patients who had AVC and underwent PD between August 2011 and December 2017 at Asan Medical Center (Seoul, Korea) were retrospectively reviewed. Patients were grouped according to surgical approach (LPD or OPD), and details of their demographics, surgical variables, postoperative outcomes, and postoperative follow-up data were collected. The following clinical, pathologic, and surgical data were collected: age at operation; sex; body mass index (BMI); American Society of Anesthesiologists (ASA) score [17]; comorbidities; preoperative biliary or pancreatic drainage; preoperative laboratory data; concurrent resection of other organs; operative time; transfusion; number of total harvested lymph nodes; tumor size; tumor differentiation; perineural invasion; lymphovascular invasion; resection margin status; tumor, node, and metastasis (TNM) stages (American Joint Committee on Cancer, 7th edition) [18]; length of hospital stay; postoperative complications; use of adjuvant therapy; recurrence; and overall survival. As for postoperative follow-up data, the duration of survival after surgery was measured from the time of surgery until death or the last visit to the outpatient department; postoperative pancreatic fistula (POPF) was defined and graded according to the recommendations of the International Study Group on Pancreatic Fistula [19]; and a clinically relevant POPF (CR-POPF) was defined as a grade B or C POPF. Postoperative complications were classified using the Clavien–Dindo classification system [20], and clinically relevant postoperative complications were defined as grade II or more severe complications. The study was approved by the Institutional Review Board of Asan Medical Center (approval number: 2018-0845).

### 2.2. Operative Procedure

The patients were informed about the advantages and disadvantages of both LPD and OPD. We presented earlier postoperative recovery and shorter operative time as the primary advantages of LPD and OPD, respectively. Whether to undergo LPD or OPD was decided by the patient and the surgeon’s preference, after discussion of both approaches. All patients provided written informed consent for their operations. Surgical procedures were mostly similar between LPD and OPD and were performed as previously described [4,5].

### 2.3. Statistical Analysis

Clinicopathologic characteristics and operative outcomes were compared between the LPD and OPD groups prior to matching. Continuous variables were analyzed using Student’s *t*-test or Mann–Whitney U test and are presented as mean ± standard deviation (SD). Categorical variables were analyzed using the χ^2^ test or Fisher’s exact test and are presented as numbers and percentages of patients. Survival rates and comparisons were estimated by the Kaplan–Meier survival curves and the log-rank test. Differences with *p* values ≤ 0.05 were considered statistically significant.

Matching between the LPD and OPD patients was performed by estimating a propensity score for each patient and matching the patients from the two groups in a 1:1 ratio. To estimate the propensity score, we used a logistic regression model using 14 variables, which were clinicopathologic characteristics that may affect perioperative and oncologic outcomes [21,22,23]. The variables consisted of 5 continuous variables (age (years), BMI (kg/m^2^), Charlson comorbidity index, tumor size (cm), and preoperative carcinoembryonic antigen (CEA) level) and 9 categorical variables (sex (male or female), ASA score (class I to V), preoperative biliary or pancreatic drainage, tumor differentiation (well, moderately, or poorly differentiated), perineural invasion, lymphovascular invasion, T stage (T1 to T4), N stage (N0 to N1) (American Joint Committee on Cancer, 7th edition) [18], and preoperative carbohydrate antigen 19-9 (CA 19-9) level) [24].

Propensity score was estimated with LPD as the dependent variable by multiple logistic regression analysis [25,26,27]. A full nonparsimonious model was developed that included all the abovementioned variables and the interaction terms between variables. Model discrimination was assessed with C statistics, and model calibration was assessed with Hosmer–Lemeshow statistics (χ^2^ = 6.1411, degree of freedom = 8, *p* = 0.6314). Propensity score matching was performed by Greedy matching using a caliper of 0.2 standard deviations of the logit of the propensity score. The absolute standardized differences were used to diagnose the balance after matching. All absolute standardized differences after matching were less than 0.1. After propensity score matching, continuous variables are presented as mean ± SD and were analyzed using a paired *t*-test. Categorical variables were analyzed using McNemar’s test or the marginal homogeneity test and are presented as numbers and percentages of patients. Survival rates and comparisons were estimated by Kaplan–Meier survival curves and Cox regression models, with robust standard errors that accounted for the clustering of matched pairs. All statistical analyses were carried out in IBM SPSS version 24.0 (IBM Corp., Armonk, NY, USA) and SAS version 9.4 (SAS Institute, Cary, NC, USA).

## 3. Results

### 3.1. Comparative Analysis between the LPD and OPD Groups—Unmatched Patients

A total of 359 patients with AVC underwent PD during the study period, of whom 76 and 283 received LPD and OPD, respectively. The demographic characteristics and preoperative factors of all unmatched patients are listed in Table 1. There were no statistically significant differences in terms of sex, BMI, ASA score, Charlson comorbidity index, or the rate of preoperative biliary (or pancreatic) drainage between the two groups.

Table 2 shows the pathologic findings of the two groups. The LPD group had a significantly lower rate of perineural invasion than did the OPD group (11.8% vs. 26.5%, *p* = 0.007). T stages were also significantly different between the two groups (*p* = 0.038).

**Figure 1:**
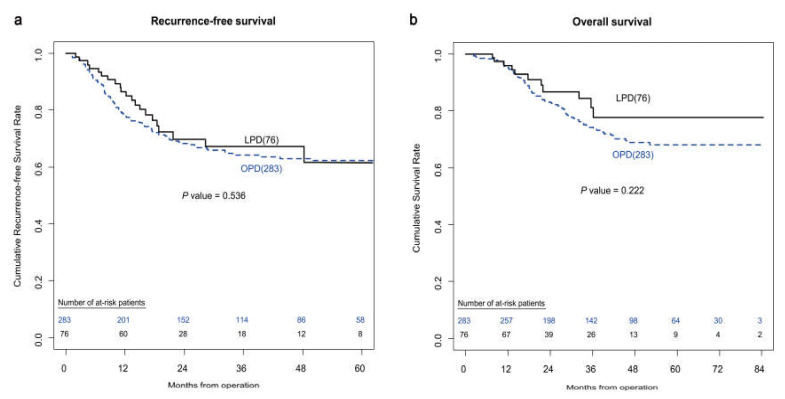
(**a**) Kaplan–Meier curves of recurrence-free survival after surgery in unmatched patients who underwent OPD or LPD. (**b**) Kaplan–Meier curves of overall survival after surgery in unmatched patients who underwent OPD or LPD.

Table 3 shows the operative outcomes and complications of unmatched patients. The LPD group had a significantly longer operation time (371 vs. 317 min, p < 0.001) and a smaller number of total harvested lymph nodes (14 vs. 16.6, *p* = 0.003). As for postoperative outcomes, the LPD group had a significantly lower rate of CR-POPF (9.2% vs. 20.1%, *p* = 0.027), a lower rate of Grade II or more severe postoperative complications (15.8% vs. 30.0%, *p* = 0.012), and shorter hospital stays (13.6 vs. 18.8 days, *p* = 0.02). There was no significant difference in Kaplan–Meier curves of recurrence-free survival (*p* = 0.536) and overall survival between the two groups (*p* = 0.222) (Figure 1).

### 3.2. Comparative Analysis between the LPD and OPD Groups—Propensity-Score-Matched Patients

**Figure 2:**
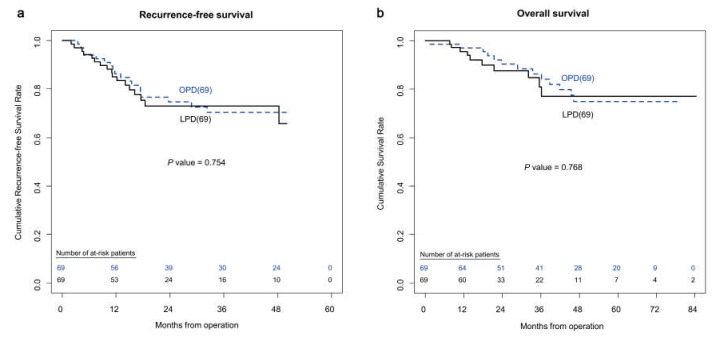
(**a**) Kaplan–Meier curves of recurrence free survival after surgery in propensity-score-matched patients who underwent OPD or LPD. (**b**) Kaplan–Meier curves of overall survival after surgery in propensity-score-matched patients who underwent OPD or LPD.

To reduce the effect of selection bias, propensity score matching was performed using the selected baseline characteristics. As a result, 69 patients from each group were matched. The two matched groups did not show any statistically significant differences in baseline characteristics and pathologic findings (Table 4). Table 5 shows the results of operative outcomes and complications of the propensity-score-matched patients. The LPD group had significantly longer operation time (400.2 vs. 344.6 min, *p* < 0.001), fewer painkiller administrations (8.3 vs. 11.1, *p* = 0.049), and a shorter duration of postoperative hospital stays (13.7 vs. 17.3 days, *p* = 0.002). In addition, CR-POPF (8.7% vs. 21.7%, *p* = 0.029) and Grade II or more severe postoperative complications (15.9% vs. 34.8%, *p* = 0.012) were less common in the LPD group. There were no significant differences in recurrence-free survival (*p* = 0.754) and overall survival between the two groups (*p* = 0.768) (Figure 2).

## 4. Discussion

“Periampullary cancer” is a nonspecific clinical term that refers to a variety of tumors, and AVC is distinct from other periampullary cancers in terms of presentation, molecular characteristics, and prognosis [28]. AVC is detected relatively early due to the appearance of jaundice and thus has a more favorable prognosis compared with other pancreaticobiliary malignancies and accounts for a large part of resectable periampullary cancers. AVC has been suggested as the most suitable for LPD among periampullary malignancies due to the relatively small size of tumors, lower possibility of vascular invasion, and higher resectability [15]. Because LPD represents a large proportion of periampullary cancers resected with PD and is suitable for LPD, it is essential to perform a comparative analysis of perioperative and oncologic outcomes between LPD and OPD in patients with AVC.

In the present study, we compared the perioperative outcomes, surrogate markers of predicting survival, and long-term survival outcomes between LPD and OPD groups in patients with AVC. We found that, compared with the OPD group, the LPD group had a higher proportion of indolent T stages and smaller proportion of perineural invasions. It is possible that surgeons may selectively include small, easily resectable tumors for LPD, thus presenting selection bias. Therefore, we conducted a propensity-score-matched analysis to minimize the influence of selection bias. After propensity score matching, there were no significant differences in the demographic and pathologic findings between the two groups.

PD involves multiple systems, and the complexity of performing three anastomoses can result in significant surgical trauma and subsequent risk of perioperative complications. Theoretically, surgical complications would be comparable between LPD and OPD because the two methods have the same resection area and reconstruction methods. However, many reports have shown that LPD is better in terms of postoperative recovery [9,10,11,12]. Likewise, our results showed improved overall morbidity, including CR-POPF, and shorter postoperative hospital stays in the LPD group. Palanivelu and colleagues performed a randomized controlled trial in patients with periampullary cancers to undergo either LPD or OPD and found that LPD resulted in shorter hospital stays [29]. We have previously reported the benefits of LPD in terms of postoperative pain, in comparison with OPD [4], in which the mean number of analgesic injections was significantly lower in the LPD group. Likewise, our current study also showed a similar result in patients with AVC in terms of the number of analgesic injections (8.3 vs. 11.1, *p* = 0.049). The lower incidence of abdominal pain in LPD is believed to be closely related to early ambulation and recovery.

Pancreaticojejunostomy is the most critical procedure during PD, and POPF resulting from failure of pancreaticoenteric anastomosis is the most common complication following PD. In the present study, the LPD group had a lower incidence of CR-POPF than did the OPD group (*p* = 0.029). We suspect that the development of bowel wall edema would have contributed to the difference in the incidence of POPF between the two groups. Marjanovic et al. showed the beneficial effects of laparoscopic surgery versus open visceral surgery in preventing bowel wall edema [30] and concluded that prevention of bowel wall edema formation is an advantage of minimally invasive surgery with respect to anastomotic healing. Hiki et al. showed that postoperative inflammation is less pronounced after laparoscopic procedures than after open surgery [31]. Less manipulation and reduced exposure of the abdominal cavity in LPD could be the main factor of lower postoperative morbidity, compared with OPD. Postoperative immune function may also be affected by the degree of surgical trauma, such as the size of the abdominal wound and exposure of abdominal organs to air. Therefore, LPD is expected to result in less systemic immune response and abnormal release of inflammatory mediators, compared with conventional open approaches. These findings shed light on why our LPD group had fewer complications than the OPD group.

Long-term survival outcomes and surrogate markers predicting survival are crucial for evaluating surgical interventions in oncology. The present study was specifically designed for AVC, and propensity score matching analysis was used in order to conduct a meaningful comparison on the oncologic outcomes between LPD and OPD.

Appropriate lymphadenectomy is crucial because elimination of a sufficient quantity of lymph nodes is helpful for improving staging accuracy and regional tumor control. In addition, curative R0 resection is regarded as the most important factor for determining a better prognosis. In the present study, both groups had similar proportions of positive resection margin and retrieved lymph nodes, and there were no significant differences in recurrent-free survival and overall survival, either. Therefore, our results show that the range of oncologic resection determines the prognosis, regardless of the surgical approach.

Because this was a retrospective study, there is a possibility of selection bias, in which patients with better baseline health and relatively milder AVC disease status may have been chosen as candidates for LPD. Although we tried to minimize such selection bias by using propensity score matching analysis, a randomized controlled study will be helpful in drawing a more definitive conclusion.

## 5. Conclusions

In conclusion, LPD for AVC showed advantages over OPD in terms of less postoperative pain, lower incidence of postoperative complications, shorter hospital stays, and acceptable oncologic outcomes, including survival. Thus, we expect LPD to be more frequently applied in patients with AVC in the future.

## Figures and Tables

**Table 1:** Demographic characteristics and preoperative factors of all unmatched patients who underwent laparoscopic pancreaticoduodenectomy (LPD) or open pancreaticoduodenectomy (OPD).

Characteristics	LPD	OPD	*p*-Value
	(*n* = 76)	(*n* = 283)	
Age, years (mean ± SD)	62.5 ± 10.3	63.7 ± 8.8	0.295
Sex, *n* (%)			0.336
Female	38 (50.0)	124 (43.8)	
Male	38 (50.0)	159 (56.2)	
BMI, kg/m^2^ (mean ± SD)	23.0 ± 2.8	23.6 ± 2.8	0.086
ASA score, *n* (%)			0.398
Class I	7 (9.2)	28 (9.9)	
Class II	60 (78.9)	235 (83.0)	
Class ≥ III	9 (11.8)	20 (7.1)	
Charlson comorbidity index (mean ± SD)	2.2 ± 1.3	2.3 ± 1.3	0.447
Preoperative biliary/pancreatic drainage, *n* (%)			0.827
No	20 (26.3)	71 (25.1)	
Yes	56 (73.7)	212 (74.9)	

BMI, body mass index; ASA, American Society of Anesthesiologists.

**Table 2:** Pathologic findings of all unmatched patients who underwent laparoscopic pancreaticoduodenectomy (LPD) or open pancreaticoduodenectomy (OPD).

Characteristics	LPD	OPD	*p-*Value
	(*n* = 76)	(*n* = 283)	
Tumor size, cm (mean ± SD)	1.8 ± 1.0	2.0 ± 0.9	0.295
Differentiation, *n* (%)			0.189
Well	27 (35.5)	79 (28.2)	
Moderate	45 (59.2)	169 (60.4)	
Poor	4 (5.3)	32 (11.4)	
Perineural invasion, *n* (%)			0.007
No	67 (88.2)	208 (73.5)	
Yes	9 (11.8)	75 (26.5)	
Lymphovascular invasion, *n* (%)			0.756
No	41 (53.9)	147 (51.9)	
Yes	35 (46.1)	136 (48.1)	
AJCC 7th T stage, *n* (%)			0.038
T1	29 (38.2)	63 (22.3)	
T2	26 (34.2)	129 (45.6)	
T3	18 (23.7)	82 (29.0)	
T4	3 (3.9)	9 (3.2)	
AJCC 7th *N* stage, *n* (%)			0.326
N0	55 (72.4)	187 (66.3)	
N1	21 (27.6)	95 (33.7)	

AJCC, American Joint Committee on Cancer.

**Table 3:** Perioperative outcomes of all unmatched patients who underwent laparoscopic pancreaticoduodenectomy (LPD) or open pancreaticoduodenectomy (OPD).

Characteristics	LPD	OPD	*p-*Value
	(*n* = 76)	(*n* = 283)	
Operative time, min (mean ± SD)	371.0 ± 90.6	317.0 ± 82.4	<0.001
Transfusion, *n* (%)			0.343
No	67 (88.2)	237 (83.7)	
Yes	9 (11.8)	46 (16.3)	
Number of harvested LN, *n* (mean ± SD)	14.0 ± 6.1	16.6 ± 8.0	0.003
Resection margin status, *n* (%)			>0.999
Negative (R0)	75 (98.7)	279 (98.6)	
Positive (R1)	1 (1.3)	4 (1.4)	
Length of hospital stay, days (mean ± SD)	13.6 ± 10.3	18.8 ± 18.4	0.02
CR-POPF, *n* (%)			0.027
No	69 (90.8)	226 (79.9)	
Yes	7 (9.2)	57 (20.1)	
Delayed gastric emptying, *n* (%)			0.453
No	72 (94.7)	261 (92.2)	
Yes	4 (5.3)	22 (7.8)	
Complications, *n* (%)			0.013
Grade 0-I	64 (84.2)	198 (70.0)	
≥Grade II	12 (15.8)	85 (30.0)	
Readmission due to complication, *n* (%)			0.093
No	65 (85.5)	260 (91.9)	
Yes	11 (14.5)	23 (8.1)	

SD, standard deviation; LN, lymph node; CR-POPF, clinically relevant postoperative pancreatic fistula.

**Table 4:** Comparison of baseline characteristics and pathologic findings between laparoscopic pancreaticoduodenectomy (LPD) and open pancreaticoduodenectomy (OPD) groups after propensity score matching.

Characteristics	Matched LPD	Matched OPD	*p-*Value
	(*n* = 69)	(*n* = 69)	
Age, years (mean ± SD)	62.8 ± 10.1	63.2 ± 8.6	0.806
Sex, *n* (%)			0.48
Female	35 (50.7)	31 (44.9)	
Male	34 (49.3)	38 (55.1)	
BMI, kg/m^2^ (mean ± SD)	23.1 ± 2.7	23.5 ± 3.3	0.367
ASA score, *n* (%)			0.8
Class I	5 (7.3)	7 (10.1)	
Class II	56 (81.2)	55 (79.7)	
Class ≥ III	8 (11.6)	7 (10.1)	
Charlson comorbidity index (mean ± SD)	2.2 ± 1.3	2.3 ± 1.1	0.667
Preoperative biliary / pancreatic drainage, *n* (%)			>0.999
No	18 (26.1)	18 (26.1)	
Yes	51 (73.9)	51 (73.9)	
Tumor size, cm (mean ± SD)	1.9 ± 1.0	1.8 ± 1.0	0.551
Differentiation, *n* (%)			0.69
Well	26 (37.7)	29 (42.0)	
Moderate	39(56.5)	38 (55.1)	
Poor	4 (5.8)	2 (2.9)	
Perineural invasion, *n* (%)			0.739
No	62 (89.9)	61 (88.4)	
Yes	7 (10.1)	8 (11.6)	
Lymphovascular invasion, *n* (%)			0.369
No	39 (56.5)	44 (63.8)	
Yes	30 (43.5)	25 (36.2)	
AJCC 7th T stage, *n* (%)			0.836
T1	26 (37.7)	31 (44.9)	
T2	25 (32.2)	22 (31.9)	
T3	16 (23.2)	14 (20.3)	
T4	2 (2.9)	2 (2.9)	
AJCC 7th N stage, *n* (%)			0.532
N0	51 (73.9)	54 (78.3)	
N1	18 (26.1)	15 (21.7)	
CA 19-9, U/ml			0.647
≤35	52 (75.4)	53 (76.8)	
35~200	13 (18.8)	13 (18.8)	
200~1000	3 (4.4)	2 (2.9)	
>1000	1 (1.4)	1 (1.5)	
CEA, ng/mL	2.3 ± 1.4	2.1 ± 1.7	0.61

BMI, body mass index; ASA, American Society of Anesthesiologists; AJCC, American Joint Committee on Cancer; CA 19-9, carbohydrate antigen 19-9; CEA, carcinoembryonic antigen.

**Table 5:** Comparative analysis of operative outcomes and complications for laparoscopic pancreaticoduodenectomy (LPD) and open pancreaticoduodenectomy (OPD) groups after propensity score matching.

Characteristics	Matched LPD	Matched OPD	*p*-Value
	(*n* = 69)	(*n* = 69)	
Operative time, min (mean ± SD)	400.2 ± 91.2	344.6 ± 80.9	<0.001
Transfusion, *n* (%)			0.796
No	60 (87.0)	59 (85.5)	
Yes	9 (13.0)	10 (14.5)	
Number of harvested LN, *n* (mean ± SD)	14.2 ± 5.9	15.5 ± 7.4	0.261
Resection margin status, *n* (%)			>0.999
Negative (R0)	68 (98.5)	68 (98.5)	
Positive (R1)	1 (1.5)	1 (1.5)	
Length of hospital stay, days (mean ± SD)	13.7 ± 10.8	17.3 ± 9.4	0.048
Number of postoperative painkiller administrations (IV or IM), *n* (mean ± SD)	8.3 ± 7.9	11.1 ± 7.5	0.049
CR-POPF, *n* (%)			0.029
No	63 (91.3)	54 (78.3)	
Yes	6 (8.7)	15 (21.7)	
Delayed gastric emptying, *n* (%)			0.109
No	65 (94.2)	59 (85.5)	
Yes	4 (5.8)	10 (14.5)	
Complications, *n* (%)			0.012
Grade 0-I	58 (84.1)	45 (65.2)	
≥Grade II	11 (15.9)	24 (34.8)	
Readmission due to complication, *n* (%)			0.808
No	60 (87.0)	61 (88.4)	
Yes	9 (13.0)	8 (11.6)	

SD, standard deviation; LN, lymph node; CR-POPF, clinically relevant postoperative pancreatic fistula.
